# Three-dimensional reconstruction of cell nuclei, internalized quantum dots and sites of lipid peroxidation

**DOI:** 10.1186/1477-3155-4-10

**Published:** 2006-10-20

**Authors:** W Robert J Funnell, Dusica Maysinger

**Affiliations:** 1 Departments of BioMedical Engineering and Otolaryngology, McGill University, 3775 rue University, Montréal, QC, H3A 2B4, Canada; 2 Department of Pharmacology & Therapeutics, McGill University, 3655 promenade Sir-William-Osler, Montréal, QC, H3G 1Y6, Canada

## Abstract

**Background:**

The purpose of the study was to develop and illustrate three-dimensional (3-D) reconstruction of nuclei and intracellular lipid peroxidation in cells exposed to oxidative stress induced by quantum dots. Programmed cell death is characterized by multiple biochemical and morphological changes in different organelles, including nuclei, mitochondria and lysosomes. It is the dynamics of the spatio-temporal changes in the signalling and morphological adaptations which will ultimately determine the 'shape' and fate of the cell.

**Results:**

We present new approaches to the 3-D reconstruction of organelle morphology and biochemical changes in confocal live-cell images. We demonstrate the 3-D shapes of nuclei, the 3-D intracellular distributions of QDs and the accompanying lipid-membrane peroxidation, and provide methods for quantification.

**Conclusion:**

This study provides an approach to 3-D organelle and nanoparticle visualization in the context of cell death; however, this approach is also applicable more generally to investigating changes in organelle morphology in response to therapeutic interventions, stressful stimuli and internalized nanoparticles. Moreover, the approach provides quantitative data for such changes, which will help us to better integrate compartmentalization of subcellular events and to link morphological and biochemical changes with physiological outcomes.

## 1. Background

Quantum dots (QDs) are increasingly being used as a complement to molecular dyes in bio-imaging applications. Compared with these dyes, QDs have two special properties: size-dependent luminescence, and broad excitation but relatively narrow emission spectra [1-3]. Different synthesis procedures and various capping and conjugation methods provide a wide array of QDs with different chemical and biological properties, including their stability in biological environments. QD stability is essential both for the quality of the imaging signal and for compatibility in live cells. Several groups have succeeded in conjugating or capping various biologically interesting ligands with QDs and have demonstrated their usefulness for different biological applications [4-14].

Despite enormous advances in biophysical, chemical and biological investigations, compatibility of QDs with live cells remains unresolved, and research in this area is still in its infancy [15-20].

QDs, like other stimuli that cause cell death, induce both subtle and robust morphological changes in several organelles, including the nucleus [21]. A type of cell death has been ascribed to the localization and extent of chromatin condensation. Depending on the kind of stressful stimulus and on its duration and intensity, nuclei become pyknotic (condensed and shrunken), hypertrophic (swollen), contorted or fragmented [22]. Several types of QDs can generate reactive oxygen species in aqueous media [16] leading to oxidative stress if the antioxidant enzymes and other cellular components cannot compensate for the insult. Gross morphological changes in cell nuclei are readily detectable by staining with commonly used fluorescent dyes, such as DAPI, DRAQ5 and Hoechst. These staining strategies are suitable for identification of nuclei, cell counting and fluorescence-activated cell sorter (FACS) analyses. Nevertheless, quantitative data from confocal microscopy images are often not provided, owing to the lack of simple and user-friendly software. Quantitative data reflecting changes in nuclear shape and volume before, during and after cell insults and pharmacological interventions are useful for correlations with cellular responses.

The nature of morphological changes and of spatial relationships among organelles can be difficult to appreciate merely by looking at two-dimensional (2-D) images, but stacks of such images can be exploited to produce three-dimensional (3-D) models using computer graphics. Depending on the type of images and on the computer algorithms used, such modelling can be more or less labour-intensive and time-consuming. Once created, these 3-D models can be used both for visualization and for quantification. In this study we provide an example of 3-D reconstruction of nuclei, of chromatin distribution and of sites of lipid peroxidation in cells undergoing oxidative stress by nanoparticles. Chromatin reorganization in cell nuclei and lipid peroxidation are critical events that can significantly impair cell function and eventually lead to cell death. Quantification of these intracellular changes is difficult to assess from 2-D images and impossible from spectrophotometric determinations.

In the present study, we describe nuclear changes induced by QD treatments in model MCF-7 and PC12 cells. We also describe effective methods for 3-D reconstruction, visualization and quantification of nuclei and QDs. We discuss two specific approaches to segmentation and 3-D reconstruction for the creation of 3-D models, and combine the approaches in order to produce composite models of the nucleus, the cell membrane and internalized QDs. We used QDs as model nanoparticles that can induce oxidative stress in order to assess: (i) morphological changes in nuclei in live cells, (ii) spatial distribution of QDs and chromatin within the cells, and (iii) lipid peroxidation that occurs as a consequence of oxidative stress.

The methods can be applied to different cell types to study changes in organelle morphologies in response to either therapeutic or nanoparticle treatments. Moreover, these methods will help to reveal fine changes in individual compartments and will provide quantitative data for such changes, thereby contributing to our understanding of the role of signal compartmentalization and strengthening the link with morphological and physiological outcomes.

## 2. Results

Numerous studies have addressed the question of intracellular changes induced by oxidative stress, including changes in nuclei, membranes, mitochondria and lysosomes [23-26]. Our previous studies showed that QDs with unprotected surfaces (without caps and shells) are relatively unstable and can cause cell death [17]. The present study illustrates new approaches to reconstructing the spatial intracellular distribution of QDs, morphological changes of the nucleus, and accompanying intracellular lipid peroxidation. The main example here is an MCF-7 cell with internalized green, positively charged QDs with cysteamine on the surface. When in serum-free medium, these cells are susceptible to QD treatment that results in significant lipid peroxidation and cell death after 24 hours (control cells: 5.7 ± 0.2%; QD-treated: 30.4 ± 1.2%; *p* < 0.05). Synthesis, characterization and concentration-dependent toxicity of these QDs were recently reported by our group [16]. In this study, our primary data consist of serial sections from live-cell imaging by confocal microscopy.

### 2.1 Morphology of nucleus

**Figure 1:**
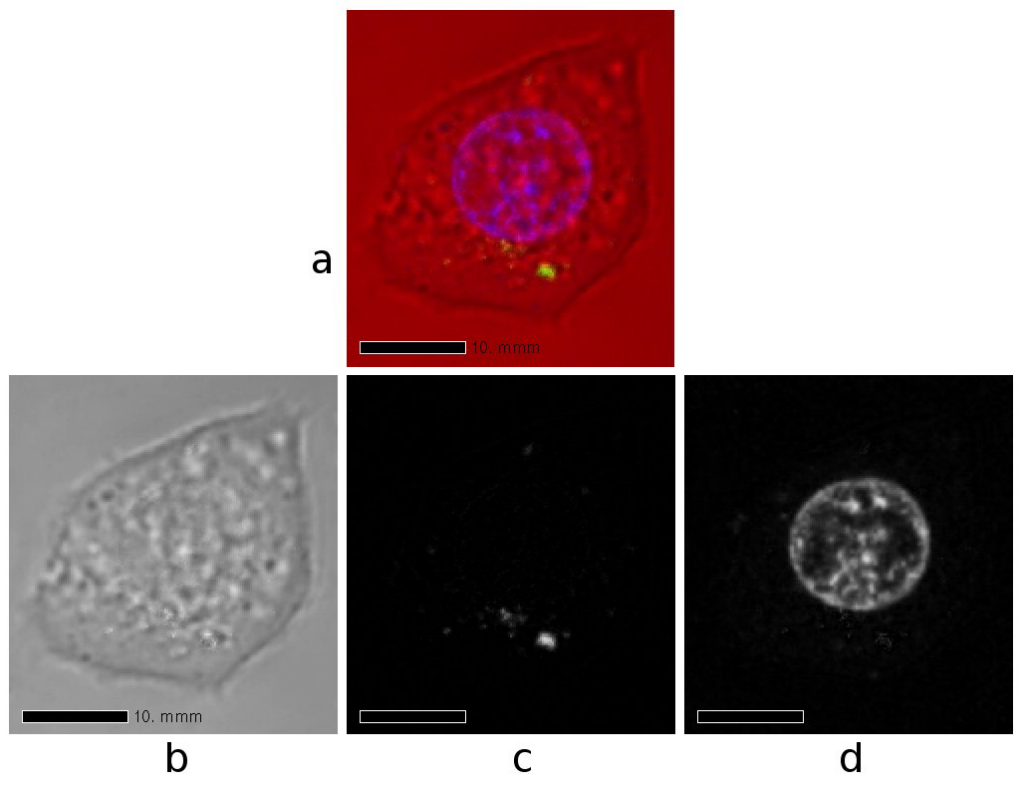
**One slice from a confocal-microscopy data set for a live MCF-7 cell**. (a) Composite of red, green and blue channels. (b) Red channel, corresponding to the confocal image without fluorescence. (c) Green channel, corresponding to the quantum dots. (d) Blue channel, corresponding to the nucleus.

The first example given here is based on an image data set containing 18 slices. The pixel (picture element) size within each image is 0.19 μm and the spacing between slices is 0.57 μm. Fig. 1 shows one particular slice. Part (b) shows the red channel, which corresponds to the confocal image without fluorescence. The outline of the cell is visible, but no special staining was used for the cell membrane. Part (c) shows the green channel, corresponding to the quantum dots, and part (d) shows the blue channel, corresponding to the nucleus.

The nucleus and cell membrane, on the one hand, have relatively simple shapes, but in the images here they do not have clean boundaries and strong contrast. A semi-automatic approach to segmentation is therefore appropriate, and in our case we use a slice-by-slice iterative boundary-fitting algorithm that is initialized and guided (and possibly corrected) manually. The procedure is described in detail under *Methods*.

The quantum dots, on the other hand, form clusters with complex shapes but have consistent intensities. An automatic threshold-based approach to segmentation is appropriate in this case and is also described under *Methods*.

**Figure 2:**
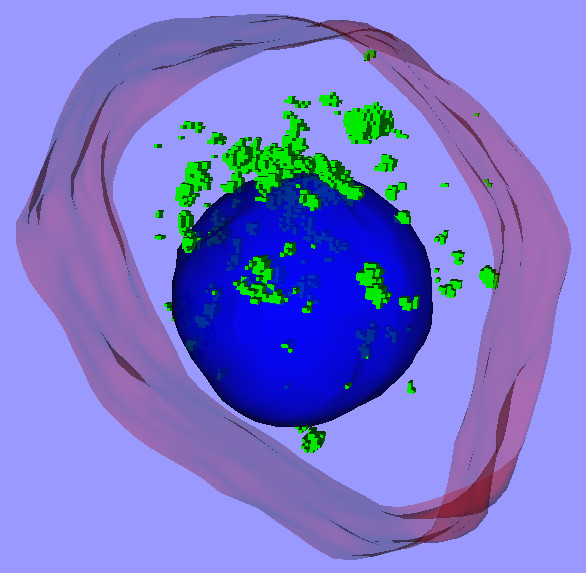
**3-D model of MCF-7 cell**. The red and blue surfaces represent the cell plasma membrane and nucleus, respectively. Each small green box represents one voxel that is considered to correspond to a cluster of quantum dots.

Fig. 2 shows the resulting 3-D model for the MCF-7 cell discussed here. The red and blue surfaces represent the cell plasma membrane and nucleus, respectively. Each small green box represents one voxel (volume element) that was above the specified threshold in the green channel and was therefore considered to correspond to a cluster of quantum dots. This kind of model can be very helpful for evaluating phenomena like 3-D shape changes due to damage caused by quantum dots, and the 3-D distributions of the quantum dots themselves.

Additional file 1 contains the complete 3-D model and can be viewed interactively with a VRML viewer. Additional file 2 is an animation created by rotating the 3-D model, for those who do not wish to install a VRML viewer.

**Figure 3:**
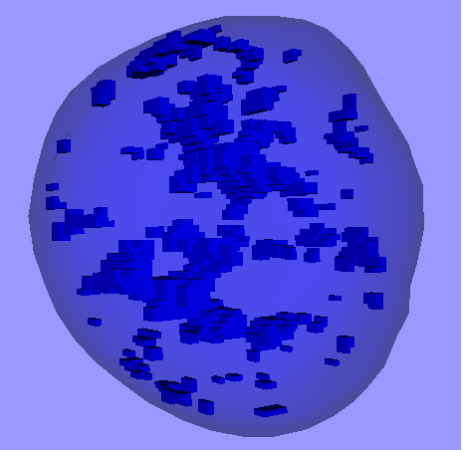
**3-D model of the nucleus of the same MCF-7 cell as in Fig. 2**. The translucent surface corresponds to the nucleus. Each small blue box represents one voxel that is considered to represent condensed chromatin.

**Figure 4:**
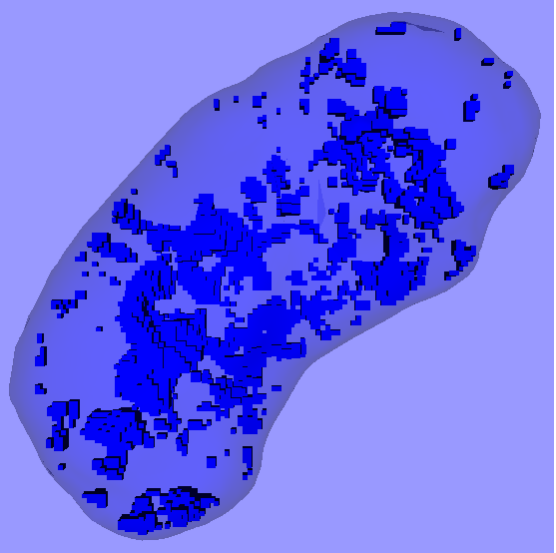
**3-D model of the nucleus of a PC12 cell**. The translucent surface and small blue boxes have the same meanings as in Fig. 3.

This technique can also be used for visualizing the distribution of condensed chromatin within the nucleus. Fig. 3 shows another model for the nucleus of the same MCF-7 cell. The overall shape of the nucleus is again shown as a translucent surface, and the chromatin with intensities above a certain threshold is shown as blue boxes. Additional files 3 and 4 contain the complete 3-D model and an animation of it. The non-uniform distribution of the chromatin is clearly visible. Fig. 4 shows a similar model for a PC12 cell [see Additional file 5].

### 2.2 Volumes of nuclei and quantum dots

We examined 2-D images of numerous nuclei and estimated the nuclear cross-sectional surface areas. For example, the changes in the cross-sectional areas of nuclei upon QD treatments were derived from 20 2-D images. There is a significant change in the average nuclear cross-sectional surface-area values in QD-treated cells (a significant decrease for pyknotic cells and an increase for hypertrophic cells compared with the controls). The cross-sectional areas are 161.7 ± 17.5 μm^2 ^for normal cells; 99.5 ± 5.6 μm^2 ^for pyknotic cells; and 202 ± 10.8 μm^2 ^for hypertrophic cells. If one assumed that the cells were spherical and that the cross-sectional areas corresponded to sections through the centres of the spheres, one could calculate the corresponding volumes with the formula

V=43π(Aπ)32.
 MathType@MTEF@5@5@+=feaafiart1ev1aaatCvAUfKttLearuWrP9MDH5MBPbIqV92AaeXatLxBI9gBaebbnrfifHhDYfgasaacH8akY=wiFfYdH8Gipec8Eeeu0xXdbba9frFj0=OqFfea0dXdd9vqai=hGuQ8kuc9pgc9s8qqaq=dirpe0xb9q8qiLsFr0=vr0=vr0dc8meaabaqaciaacaGaaeqabaqabeGadaaakeaacqWGwbGvcqGH9aqpdaWcaaqaaiabisda0aqaaiabiodaZaaaiiGacqWFapaCdaqadaqaamaalaaabaGaemyqaeeabaGae8hWdahaaaGaayjkaiaawMcaamaaCaaaleqabaWaaSaaaeaacqaIZaWmaeaacqaIYaGmaaaaaOGaeiOla4caaa@3A12@

The above areas would thus correspond to volumes of 1547 μm^3 ^(normal), 747 μm^3 ^(pyknotic) and 2160 μm^3 ^(hypertrophic), respectively. These estimates, however, depend critically on the two assumptions, neither of which is likely to be valid. The assumption of sphericity may be applicable to normal MCF-7 nuclei, but it is not applicable to abnormally shaped ones and it is not at all applicable to PC12 cells, for example, which have irregularly shaped nuclei even in the normal condition. More sophisticated stereological methods are available to obtain unbiased volume estimates for populations of objects [27]. To estimate the volumes of individual nuclei, one can compute them from reconstructed 3-D surfaces, as discussed in *Methods*. For example, for the 3-D model of an MCF-7 cell discussed above, the volume of the nucleus was computed to be 1180 μm^3^.

Quantification of QDs is almost impossible from single 2-D images since the distribution of fluorescence is extremely variable from one slice to another. It is therefore necessary to use the information from all slices. Across all of the slices in this data set, the total number of voxels corresponding to quantum dots is 2856; given the pixel size and slice spacing, the volume of one voxel is 0.02 μm^3^, so the total volume for the quantum dots is 58.8 μm^3^, or about 5% of the volume of the nucleus.

**Figure 5:**
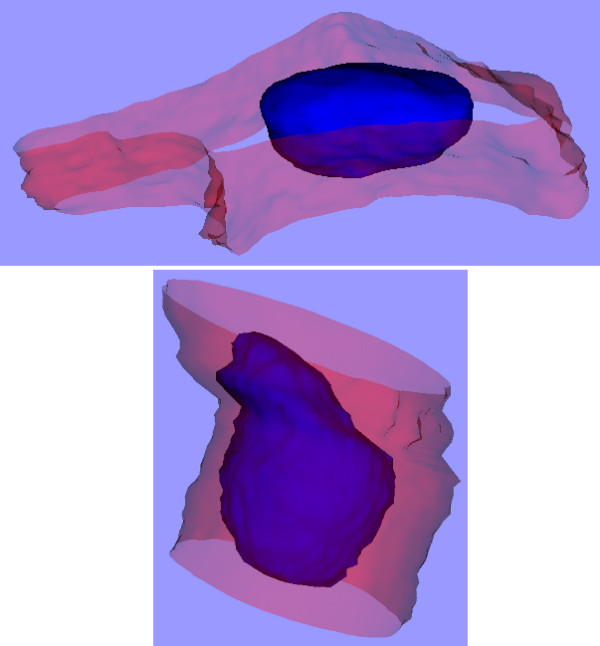
**3-D models of two control MCF-7 cells**. The surface colours are as in Fig. 2.

For comparison we show here two other examples, both again for MCF-7 cells. In Fig. 5 [see Additional files 6 and 7], the image on the top is of a control cell. The data set contains 20 slices with a spacing of 0.41 μm, and a pixel size of 0.19 μm again. The nucleus volume is 1440 μm^3^. The image on the bottom (not to the same scale) is of another control cell. This data set contains 30 slices with a slice spacing of 0.61 μm, and again a pixel size of 0.19 μm. The nucleus volume is 1530 μm^3^. The nucleus volumes of these control cells are 22% and 30% larger, respectively, than that of the QD-treated MCF-7 cell presented above.

### 2.3 Lipid peroxidation

**Figure 6:**
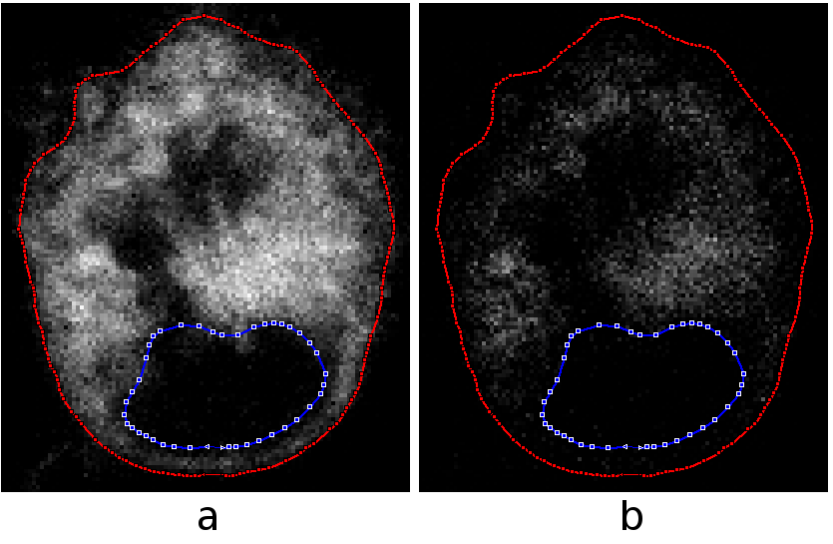
**One slice from a confocal-microscopy data set for a live MCF-7 cell with BODIPY fluorescent dye**. The red and blue lines represent the cell membrane and nucleus as segmented interactively. (a) Red channel (unoxidized lipids). (b) Green channel (oxidized lipids).

Proteins, DNA and lipids are susceptible to reactive oxygen species and they can be peroxidized under oxidative stress conditions. We assessed the extent of lipid peroxidation in cells exposed to QDs by using BODIPY C_11 _@581/591. This fluorescent dye emits red fluorescence when lipids are unoxidized and green when they are oxidized. A ratiometric approach using spectrofluorometry clearly showed that QD treatment causes lipid peroxidation. (The ratio between the red and green was significantly decreased in QD-treated cells: control = 1553.9 ± 270.2, QD-treated = 512.3 ± 49.9, *p *< 0.05.) However, given that the spectrofluorometric approach does not provide spatial information, we used the same dye to generate images with confocal microscopy. An example is shown in Fig. 6, in which parts (a) and (b) show the red and green channels, respectively. The red and blue lines represent the cell membrane and nucleus as segmented with the interactive technique described under *Methods*.

**Figure 7:**
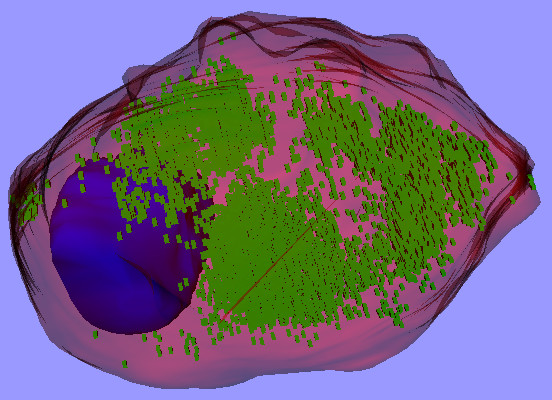
**3-D model of the MCF-7 cell shown in Fig. 6**. The surface colours are as in Fig. 2. The small green boxes represent voxels for which the green channel (oxidized lipids) had intensities above a specified threshold.

Three-dimensional models provide means for quantification and can also show the spatial distributions of intracellular lipid oxidation. Fig. 7 shows a view of a 3-D model [see Additional file 8] in which the cell membrane and nucleus are based on the segmentation shown in part by the red and blue lines in Fig. 6, and the voxels corresponding to the green channel (oxidized lipids) are displayed with boxes based on a threshold.

From Fig. 6(a) and (b) and  it is clear that there is more unoxidized lipid than oxidized. To quantify the observation, one can compute the total intensity in each channel, as described under *Methods*. In the cell slice shown in Fig. 6, there is a total intensity of 671 × 10^3 ^in the red (unoxidized) channel and only 131 × 10^3 ^in the green (oxidized) channel, a ratio of about 5.1. Summing over all slices, one obtains for this cell a total red intensity of 16.5 × 10^6 ^and a total green intensity of 2.89 × 10^6^, a ratio of about 5.7. The similarity of the ratios suggests that this slice is typical. For a second cell from the same data (not shown), the total intensity across all slices is 19.3 × 10^6 ^for the red channel and 1.83 × 10^6 ^for the green, for a ratio of about 10.5.

**Figure 8:**
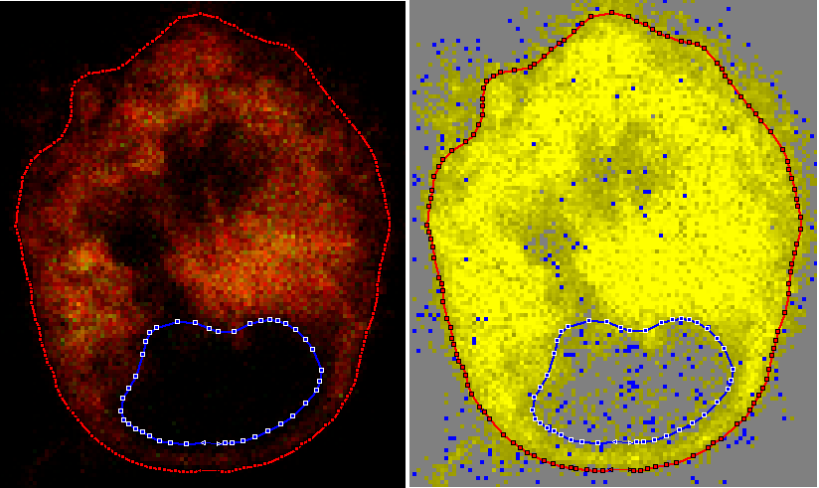
**Composite images of the same slice as in Fig. 6**. (a) Additive combination of red and green channels. (b) Subtractive combination of red and green channels, as described in the text.

To visualize the distribution of relative intensities, one can view the additively combined red and green channels in the conventional way, as in Fig. 8(a), but this is difficult to quantify. An alternative is to display the differences between the red and green channels as described under *Methods*. The result is shown in (b). The pixels are shown as grey when the intensity difference is zero, as shades of yellow when the red intensity is greater than the green intensity, and as shades of blue when the opposite is true. The dominant bright yellow and the sparse scattering of blue confirms the impression obtained from viewing the separate channels in (a) and (b) of Fig. 6.

## 3. Discussion

In this study we present a versatile, simple and suitable approach to 3-D reconstruction of intracellular QD location and of changes in nuclear morphology and lipid peroxidation as a consequence of oxidative stress [16,17,28-33]. The approach we present here is applicable to any cell type and to any pharmacological or genetic manipulation that leads to morphological and/or biochemical change. It is also applicable to much larger anatomical structures [34,35] and to different organelles that play a role in cell death [18].

Many approaches are available to create and share 3-D models. Although 3-D models can be produced by the free-form creation of arbitrary shapes, in this paper we are considering the case in which the form of the model is derived from 3-D imaging data consisting of a stack of aligned images. Such models are often based on X-ray computed tomography (CT) and magnetic resonance imaging (MRI), but here we are considering confocal optical-sectioning microscopy. Compared with CT and MRI data, optical sectioning often results in fewer slices and in a slice thickness that is greater than the within-slice pixel size.

The geometry of the model is derived by segmenting the images, that is, by determining which parts of the images correspond to the structures of interest. The segmentation process can vary from purely manual 2-D (one slice at a time) to fully automatic 3-D. In this paper we demonstrate the use of two different approaches, depending on the nature of the images and of the structures to be segmented.

For images that do not have clean boundaries and strong contrast, a semi-automatic approach is appropriate. In this case we use a slice-by-slice iterative boundary-fitting algorithm that is initialized and guided (and possibly corrected) manually. This technique can become laborious and is best used either when the shapes to be segmented are relatively simple or when the image quality is simply not good enough to permit a more automatic technique. As described under *Methods*, a number of parameters can be adjusted to modify the behaviour of the algorithm. The best parameter settings to use will depend on the nature of the images and even on the different natures of specific structures within the images. Once a set of parameters has been established, it can be left unchanged while a number of data sets are being analyzed if it is important to maintain consistency for the sake of quantification and comparison.

**Figure 9:**
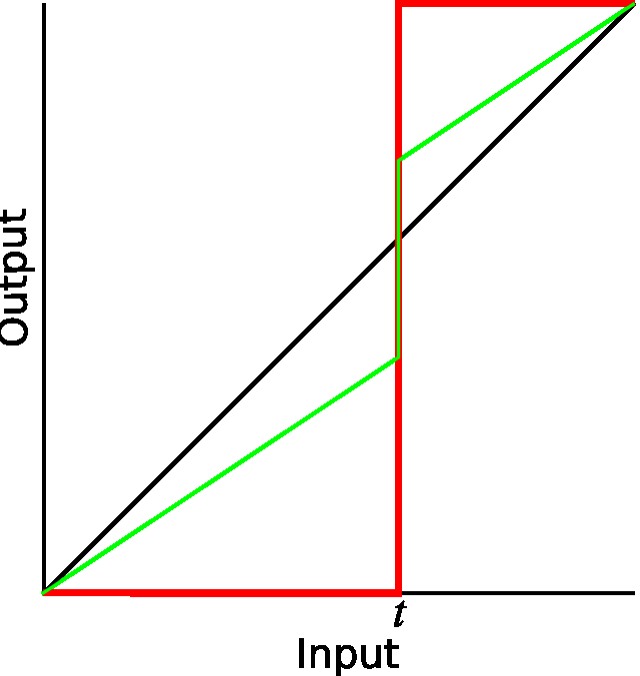
**Effect of threshold-strength parameter**. Effect of threshold-strength parameter on grey levels passed to segmentation algorithm, as discussed in text. Black line corresponds to strength = 0; red line corresponds to strength = 1; green line corresponds to an intermediate value.

One particular parameter used to guide the segmentation algorithm here is *threshold strength*. Typically in image-segmentation work, if a threshold is used at all, it is used to completely determine the segmentation: pixels or voxels whose intensities are above the threshold are considered to be part of the structure, and those whose intensities are below the threshold are not. This can lead to very rough boundaries even for smooth structures because of noise in the image-acquisition process. The fact remains, however, that a threshold can often be a good visual indication of where the boundaries should be. In our approach we assign a strength parameter to the threshold, as illustrated in Fig. 9. If the strength is zero (black line), the threshold is only for visualization by the user and has no effect on the segmentation algorithm. If the strength is one (red line), the threshold completely controls the image information used by the algorithm. Usually we use an intermediate value (green line), so the boundary-seeking behaviour of the algorithm is influenced by the threshold but can also be influenced by the shades of intensity around the boundary.

The lower the quality of an image and the greater the required precision, the more time-consuming an interactive segmentation technique will be. In the images shown here, the most challenging segmentation is that of the cell membrane. If the exact overall shape of the cell required careful study, however, a special stain could be used for that purpose, thus increasing the boundary contrast and greatly facilitating the segmentation process.

The second segmentation technique used here is fully automatic 3-D threshold-based segmentation. For structures that have relatively good image contrast, well-defined edges and uniform intensities, it is feasible to use automatic threshold-based segmentation algorithms. Such automatic segmentation is particularly desirable for structures with very irregular boundaries or for large numbers of small structures, given that in both cases interactive segmentation would be extremely tedious.

Regardless of which segmentation technique is used, if the sizes of the segmented structures are to be quantified then it is obviously desirable that the boundaries be correctly identified. This applies equally to 2-D and 3-D methods. It is often difficult, however, to determine what is 'correct' since boundaries are often not sharply delineated. In some situations it might be possible to compare results with other imaging modalities, such as transmission electron microscopy (TEM) serial sections, but every technique has its own question marks. In the case of TEM, for example, the boundaries may be very sharp but the distortion due to fixation and other processing is difficult to determine.

For many purposes, absolute accuracy is not as important as consistency, as when comparing the sizes of one population of structures with those of another population. As long as the imaging parameters are kept the same, segmentation consistency can be obtained with fully automatic threshold-based segmentation simply by keeping the threshold unchanged. Even with semi-automatic techniques, consistency can be obtained by keeping the various segmentation parameters unchanged once they have been determined, as mentioned above, and by limiting subsequent manual intervention to simple tasks like the selection of regions or structures to be processed in different data sets.

An important feature of the type of images used here is that they involve two or three independent colour channels. In other biomedical imaging applications, the images are generally either monochromatic (e.g., X-ray CT and MRI) or 'real' colour (e.g., histology). In the case of real colour, the images are most often represented numerically as red, green and blue channels but are conceptually composed of hue, lightness and saturation. In the present case, however, the red, green and blue channels actually represent independent sources of information (e.g., different laser frequencies). This implies that the segmentation should be carried out one channel at a time and that the software should be able to display and process the three channels either individually or in various combinations.

Once a set of images has been segmented in some way, different techniques are available for creating 3-D surface models. The two techniques presented here, triangulation between contours and simple labelling of voxels, are complementary. The resulting 3-D models can be presented as still images or as movies, but they are much more informative if they can actually be manipulated interactively in 3-D. The use of the VRML97 standard file format for interchanging 3-D models via the World Wide Web allows models to be shared with other researchers, who can easily install free 3-D viewers on their own computers without the need for any special hardware.

The X3D standard is an 'enhanced successor to VRML' but VRML remains useful 'while developers update their products to support X3D' [36].

## 4. Conclusion

The approach presented here is suitable for 3-D reconstructions of multiple intracellular events that include both morphological and biochemical changes in different organelles. The results show how 3-D reconstructions can provide quantitative information for simultaneous intracellular morphological changes (e.g., nuclear shrinkage or expansion) and biochemical changes (e.g., lipid peroxidation) occurring in stressed cells. The approach is applicable to any cell type and any event discernible with fluorescent markers (e.g., Lysotrackers to reveal lysosomal swelling, Mitotrackers for mitochondrial morphology or JC-1 for changes in mitochondrial potential) detectable by confocal microscopy.

## 5. Methods

### 5.1 Cell preparation

All studies involving cell cultures were approved by the Biohazards Committee of McGill University under the conditions certified by the committee and recommended by the ATCC (American Type Culture Collection).

Quantum dots were prepared and characterized as described previously [16].

Rat pheochromocytoma (PC12 cells) and human breast cancer (MCF-7) cells were cultured (37°C, 5% CO_2_) in RPMI 1640 medium containing 10% fœtal bovine serum (FBS) (Gibco, Burlington, ON, Canada). RPMI 1640 medium was phenol-red free and contained 1% penicillin-streptomycin. For spectrofluorometric and colorimetric assays, cells were cultured in 24-well plates (Sarstedt, Montréal, QC, Canada) at a density of 105 cells/cm^2^.

One hour prior to treatments, medium containing serum was aspirated, and cells washed with serum-free medium. Fresh serum-free medium was added to all wells except to control cells grown in 10% FBS to account for changes in cell morphology, cell number and metabolic activity caused by the serum withdrawal.

QD solutions (5 or 10 μg/mL) were prepared from the stock (2 mg/mL) by dilution in serum-free cell-culture medium. Cells were incubated with QDs for maximum of 24 h before biochemical analysis or live-cell imaging.

### 5.2 Cell viability

Following the QD treatments, cells were stained with Hoechst 33342 (10 μM, 1 hour). The number of nuclei was determined by counting all fluorescent nuclei, regardless of their shapes, with triplicate measurements being done per condition as a minimum. Alternatively, the fluorescence intensity was measured by spectrofluorometric readings with the SpectraMax Gemini XS microplate spectrofluorometer (Molecular Devices Corporation, USA). Cell number was determined from the linear portions of calibration curves (RFI for Hoechst versus cell number). Data were analyzed with the SOFTmax Pro 4.0 programme. All values are presented in percentages relative to the respective serum-negative control.

### 5.3 Lipid peroxidation

Cells were treated with the fluorescent dye BODIPY^® ^581/591 C_11 _(BODIPY-C_11_, Molecular Probes), which inserts into cell membranes and allows for quantitative assessment of oxidized versus unoxidized lipids by fluorescing green or red, respectively, and analyzed either by spectrofluorometry or by confocal microscopy. Cells were stained for 30 min with a 10 μM solution of BODIPY-C_11 _prior to QD treatment. After the QD treatment, spectrofluorometric samples were prepared as follows: lipids were extracted from the cells according to the Folch method by incubating twice with a mixture of chloroform and methanol (2:1 [v/v]). After extraction, 0.2 volumes of 0.9% NaCl solution were added and the chloroform-containing phase was collected. After evaporation of the chloroform and dissolving of the lipids in isopropanol, spectrofluorometric readings were taken with the SpectraMax Gemini XS microplate spectrofluorometer (Molecular Devices Corporation, USA). Data were analyzed with the SOFTmax Pro 4.0 programme. All values are presented as normalized means ± SEM relative to the respective serum-negative control (taken as 100%). Values were considered significant where *p *< 0.05.

### 5.4 Confocal microscopy

Images were acquired with a Zeiss LSM 510 NLO inverted microscope. Cells were grown on 8-well chambers (Lab-Tek, Nalge Nunc International, Rochester, NY, USA). QDs were added to designated wells and the cells were incubated for 24 hours. Nuclei were stained with Hoechst 33342 (10 μM, 30 min—1 h, Molecular Probes; λ_ex _350 nm, λ_em _461 nm) and analyzed by 2-photon imaging (Ti:Sa laser set to pulse at 800 nm and BP 390–465 IR filter). Lipid peroxidation was assessed by staining with BODIPY-C_11 _(Molecular Probes) and the shift from red to green was monitored with a HeNe laser (543 nm, LP 560 nm filter) and an argon laser (488 nm, LP 520 nm filter). No background fluorescence of cells was detected under the settings used. Images were acquired at resolutions of 512 × 512 and 1024 × 1024. In all the imaging experiments, the number of averages was 4. Scan size was 146.2 μm × 146.2 μm.

### 5.5 Statistical analysis

Data were analyzed with SYSTAT 10 (SPSS, Chicago, IL, USA). Statistical significance was determined by Student's t-test, one-way ANOVA followed by multiparametric Dunett's post-hoc test, or two-way ANOVA. Differences were considered significant where *p *< 0.05.

### 5.6 Interactive image segmentation

#### 5.6.1 Introduction

**Figure 10:**
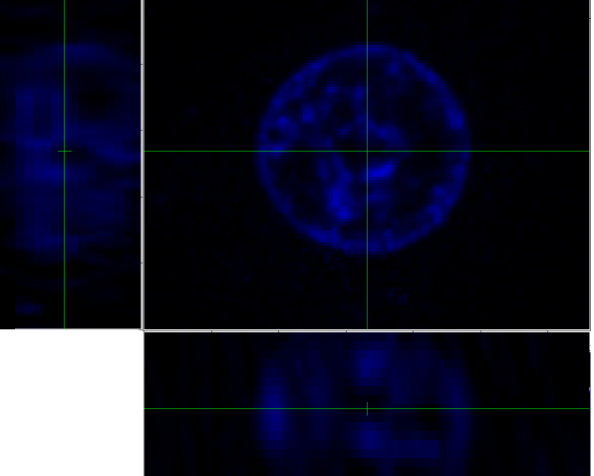
**Blue-channel image for a cell nucleus imaged with blue fluorescence**. The large image is one of the optical-sectioning slices (*x*-*y *plane). The images to the left (*z*-*y *plane) and below (*x*-*z *plane) are perpendicular sections through the stack of slices.

In this section we describe our approach to interactive image segmentation, illustrating it for one particular data set. Fig. 10 shows the original blue-channel image for a cell nucleus imaged with blue fluorescence. The large image is one of the optical-sectioning slices, in what we shall refer to as the *x*-*y *plane. The images to the left and below are perpendicular sections through the stack of slices; the one on the left represents the *z*-*y *plane and the one below represents the *x*-*z *plane.

#### 5.6.2 Choice of threshold

Because the image contrast is quite low, we manually set a threshold to guide the segmentation process. The choice of threshold is subjective, and will affect the shape and volume of the resulting 3-D model.

**Figure 11:**
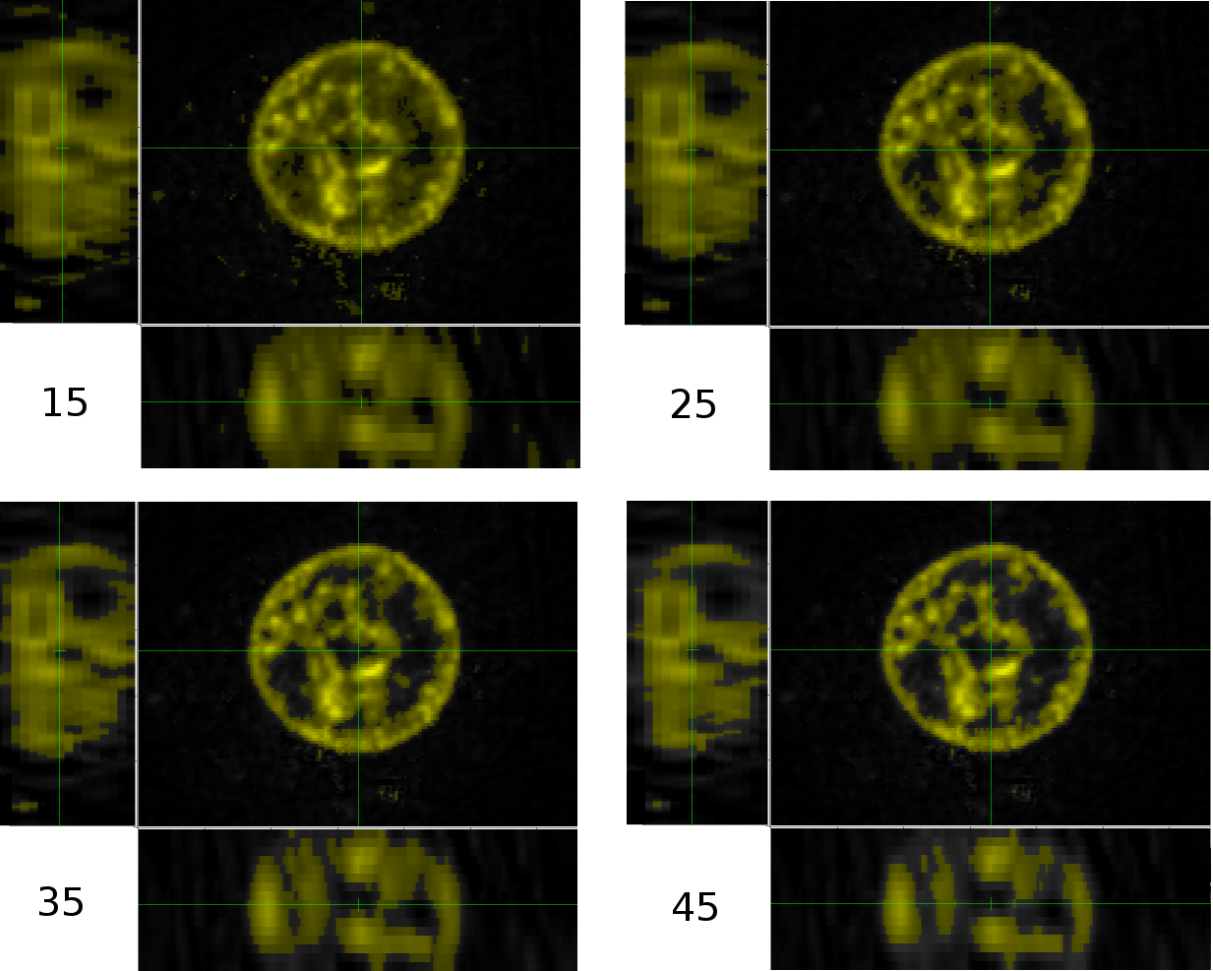
**Same blue-channel data as shown in Fig. 10**. Threshold values of 15, 25, 35 and 45.

The four images in Fig. 11 show the same blue-channel data but with four different threshold values. On a scale from 0 to 255, the thresholds are 15, 25, 35 and 45. Pixels whose intensities in the blue channel are greater than or equal to the threshold are coloured yellow and brightened somewhat.

For all four threshold values shown, the circular outline of the nucleus is well defined and continuous in the *x*-*y *image, but the interior is far from uniform. Even for the lowest threshold value, the interior of the nucleus still contains regions of unselected voxels. For the higher thresholds, a few voxels are selected that are clearly outside the nucleus, and the number increases as the threshold is decreased.

The side views change considerably as the threshold is changed. At a threshold of 15, significant numbers of selected voxels are clearly outside the nucleus. At a threshold of 45, in the left-hand image the outline of the nucleus has an enormous concavity that presumably does not correspond to the real shape of the nucleus, and in the bottom image the nucleus appears to be divided into several large chunks.

**Figure 12:**
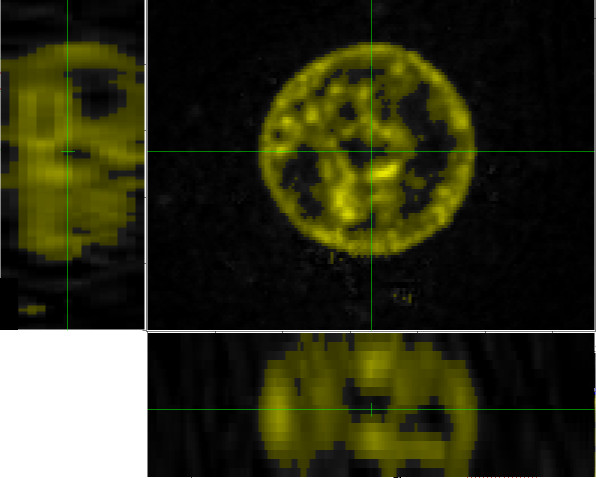
**Same blue-channel data as shown in Figs. 10 and 11**. Threshold value of 30.

A reasonable choice of threshold would seem to be between 25 and 35. Fig. 12 shows a threshold of 30, which is the value that we shall use here.

#### 5.6.3 Segmentation

As mentioned above, an iterative algorithm with manual intervention is used for the segmentation process. The algorithm is an implementation [37] of 'discrete dynamic contours', or 'snakes' [38]. The software, *Fie*, was developed locally and can be downloaded from the Web [39]. It is written in Fortran and can currently be run under GNU/Linux for Intel-compatible and HP Alpha processors, and under Microsoft Windows.

The general notion of the snake algorithm is that nodes are generated along the contour to be fitted, and then 'forces' are computed at each node: external forces that depend on the image and drive the contour towards features in the image, and internal forces that depend on the shape of the contour and maintain some degree of smoothness. The forces are computed iteratively and drive the contour towards some equilibrium position. The software contains many user-settable parameters, including relative weightings for the external and internal forces and for additional 'pressure' forces; a choice between normal image gradient and 'gradient vector flow'; desired vertex spacing, down-sampling ratio and blurring; whether and to what extent thresholds are used to influence the snake; and damping and time-step parameters that influence the evolution of the iterative process. These parameters can all be used to adapt the algorithm to the nature of the image.

**Figure 13:**
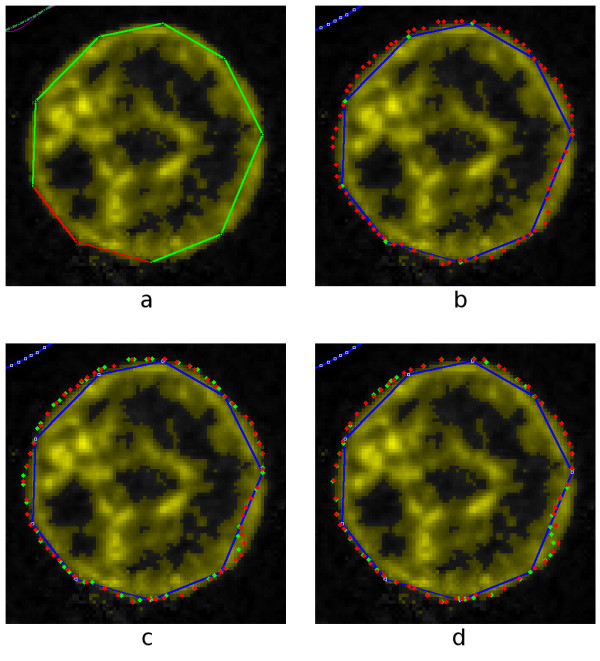
**Steps in iterative fitting of boundary**. (a) Initial manual segmentation with only nine points. (b) After 100 iterations of snake algorithm. (c) After 200 iterations. (d) After 300 iterations.

In the example shown here, we started with slice 9 of 18 slices by doing a very fast and crude manual segmentation with only nine points (Fig. 13a).

We then used the iterative snake algorithm to refine the manual segmentation. Part (b) of the figure shows the original boundary (blue lines with green nodes) and the boundary after one set of 100 iterations (red nodes). Such a set of iterations is invoked with a single mouse click or keystroke and takes only a fraction of a second.

The new boundary fits the outline of the nucleus very well except in the lower right quadrant, where the algorithm was confused because the manual boundary passed close to an internal boundary. Parts (c) and (d) of Fig. 13 show how the boundary nodes approached the desired boundary after 100 and 200 more iterations, respectively. After a few hundred more iterations (a few more keystrokes), the boundary was close to the nucleus outline everywhere.

**Figure 14:**
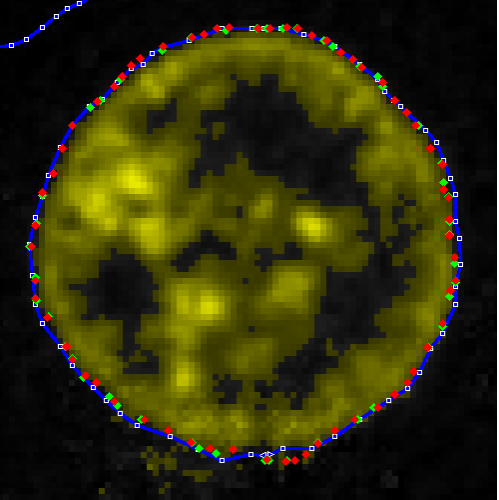
**Example of copying boundary**. Example of satisfactory boundary obtained after copying boundary from previous slice and iterating 200 times.

Once a boundary has been well defined in one slice, it can be copied and pasted into another slice and iteratively fitted to the new image. Fig. 14 shows the result of having pasted the contour from slice 9, obtained as described above, onto slice 10 and then having iterated the snake algorithm 200 times, resulting in the new boundary defined by the red nodes.

**Figure 15:**
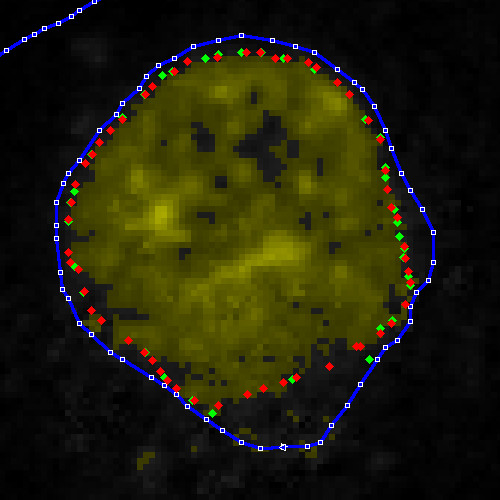
**Example of need for manual intervention**. Example of need for manual intervention after boundary was copied from previous slice, as described in the text.

The copy, paste and iterate procedure can be repeated from slice to slice very quickly, as long as the desired boundary does not change too much. Fig. 15 shows an example in which the boundary changes greatly from slice to slice, requiring manual intervention. The blue line is the boundary from the previous slice. When it became obvious that the snake algorithm could not bridge the large gap at the bottom of the nucleus, the manual-editing function was used to quickly delete a number of nodes from the boundary and insert a single node halfway across the resulting gap. The result is shown by the green nodes. A single set of 100 iterations then sufficed to produce a good boundary all the way around, shown by the red nodes.

**Figure 16:**
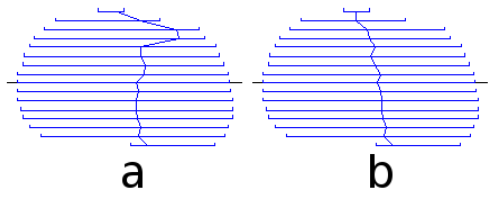
**Side view of segmentation of example nucleus**. (a) Before adjusting starting points. (b) After adjusting starting points.

The copy, paste and fit procedure was repeated until the last slice. We then returned to the starting slice and worked backwards to the first slice by using the same copy, paste and fit sequence. Fig. 16(a) shows a side view of the resulting segmentation. The line running from top to bottom shows the positions of the starting points of the contours on every slice. The starting points became offset from one another as a result of the editing and drastic refitting required when the boundary shape and size varied greatly. Large starting-point offsets, like those between the second and third slices from the top, and between the fourth and fifth slices, would cause problems for the subsequent 3-D surface generation process, so an automatic starting-point alignment algorithm was used. The result is shown in Fig. 16(b).

**Figure 17:**
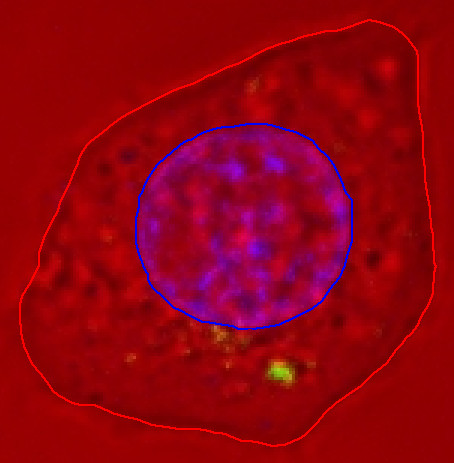
**Colour image of one slice**. Channel colours are as in Fig. 1. Blue line indicates nucleus as segmented using blue channel; red line indicates cell membrane as segmented using red channel.

For boundaries with less contrast, such as a cell membrane for which no special staining has been used, the snake algorithm can still be used but more manual intervention is likely to be required and the actual position of the boundary is less certain. Fig. 17 shows the cell as the red channel in one particular slice. The blue line indicates the nucleus as segmented using the blue channel, as discussed above, and the red line represents a segmentation of the cell membrane.

### 5.7 Threshold-based image segmentation

**Figure 18:**
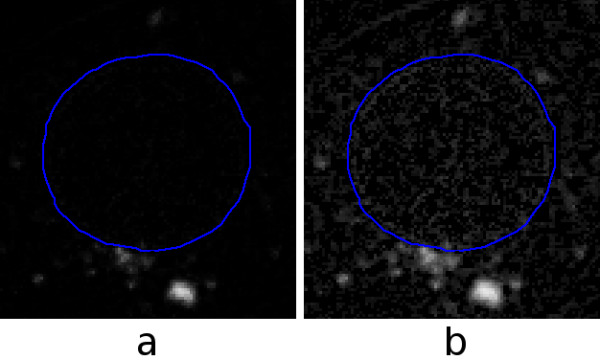
**Quantum-dot channel in one slice**. (a) Gamma = 1.0. (b) Gamma = 0.5.

This section describes automatic 3-D threshold-based segmentation. As an example for which automatic thresholding works well, Fig. 18(a) shows the quantum-dot channel in one particular slice. The blue line is the segmented outline of the cell nucleus. There is one large bright area and some fainter areas. To show the fainter areas more clearly, Fig. 18(b) presents the same image with a non-linear brightness scale, corresponding to a gamma value [[40], p. 564] of 0.5 (rather than the original 1.0). Some fainter clusters are now also visible, as well as a fairly uniform background scattering of pixels that are not quite black. The segmentation technique described above for the cell nucleus would be extremely difficult and time-consuming if applied to the many clusters of pixels corresponding to quantum dots.

**Figure 19:**
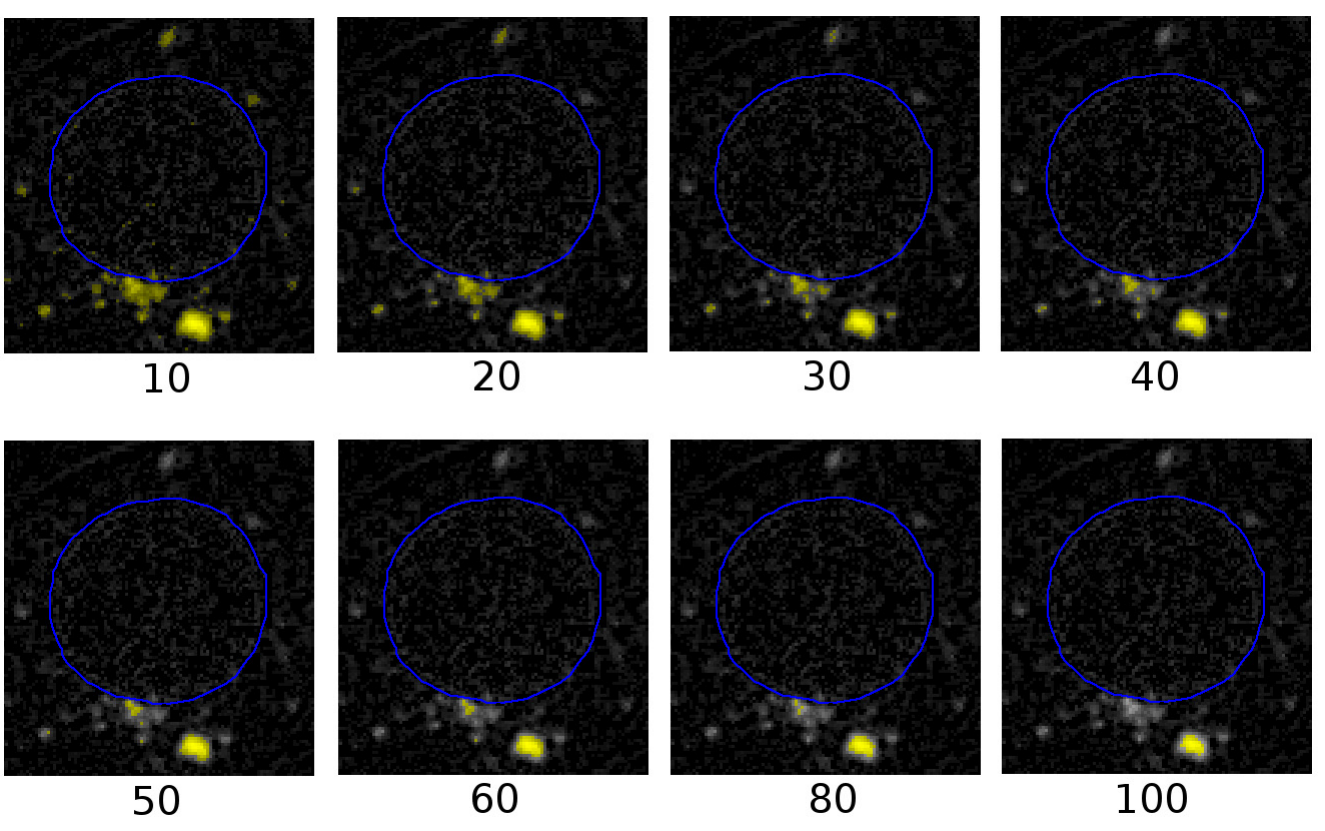
**Effects of different thresholds**. Same image as in Fig. 18 with various thresholds applied.

Once again it is necessary to choose a threshold, in this case for selecting which pixels will be considered to belong to quantum dots. In Fig. 19, various thresholds from 10 to 100 (on a range of 0–255) are illustrated. Any pixels whose intensities are greater than or equal to the threshold are again coloured yellow and brightened somewhat.

It seems clear that a threshold of 10 is too low and a threshold of 100 is too high. If the cluster of pixels above the nucleus is considered to be quantum dots, the threshold should be taken as 20 or 30. We use a value of 30 here.

Once a threshold is chosen, each voxel with an intensity value greater than or equal to the threshold will be considered to belong to the structure. Our *Fie *programme includes a function that allows all voxels at or above the threshold to be flagged with a single keystroke for the entire image volume.

### 5.8 Three-dimensional model creation and distribution

#### 5.8.1 Contour-based surface reconstruction

**Figure 20:**
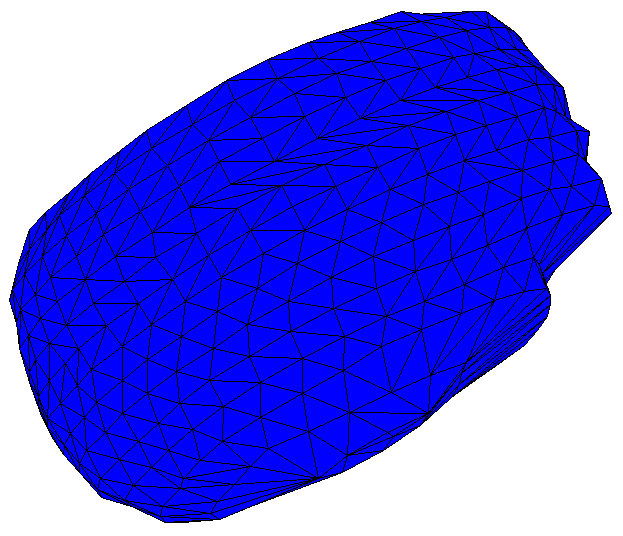
**Surface generated for nucleus**. View of 3-D surface generated for nucleus.

Once the boundary of a structure has been defined as a contour in every slice, a 3-D surface can be created by generating sets of triangles between the contours. This was done here using our locally developed *Tr3 *programme, downloadable from the same location as *Fie*. It accepts a user-defined resolution parameter that determines how many triangles to generate, that is, how fine a surface triangulation is desired. An optimization algorithm is then used to decide how to generate the triangles [41]. Figure 20 shows one view of the 3-D surface generated for the nucleus in the example discussed here, illustrating that the surface is composed of triangles.

**Figure 21:**
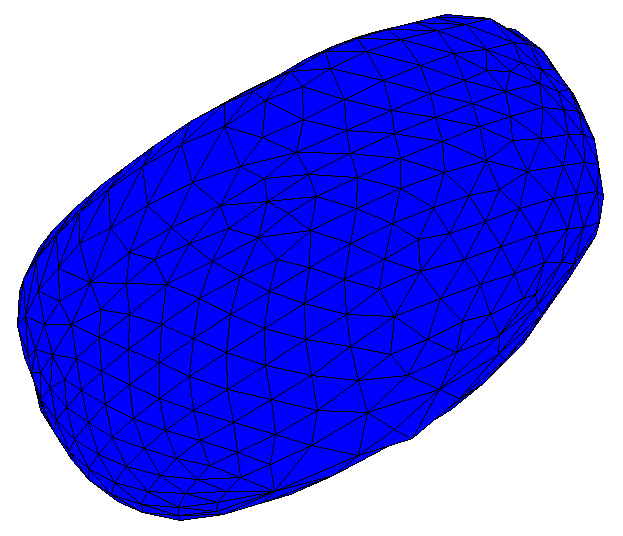
**Smoothed surface generated for nucleus**. View of 3-D surface generated for same nucleus as in Fig. 20, after smoothing.

Surfaces reconstructed in this way are generally not very smooth, at least partially as a result of image noise and segmentation errors. To some extent the segmentation errors can be reduced by investing more effort in the segmentation, but the effects of noise remain. If the biological structure is expected to be smooth, then one can simply apply a final automatic smoothing function. Such smoothing will tend to shrink structures unless special care is taken. The reduced-shrinkage algorithm used in *Tr3 *is that of Taubin [42]. Figure 21 shows a smoothed version of the surface shown in Figure 20. Since even this smoothing algorithm may cause some change in volume, the values given for organelle volumes here are based on the original unsmoothed surface triangulations.

#### 5.8.2 Voxel-based 3-D models

For the 3-D surface reconstruction of models derived from thresholded voxels, two popular choices are the patented *marching cubes *algorithm [43] and related but perhaps more elegant (and unpatented) algorithms based on tetrahedra rather than cubes [44,45]. Here, however, we have chosen simply to create a small 3-D box for each voxel that qualifies. This emphasizes the underlying voxel structure in the resulting 3-D model, which is particularly appropriate and feasible for confocal microscopy and optical sectioning, where the number of slices tends to be fairly small and the voxels relatively large. The *Tr3 *programme generates a separate 3-D box for each voxel that has been flagged for display.

#### 5.8.3 Viewing and sharing 3-D models

*Tr3 *can generate the 3-D model in several different forms, for different purposes. The form of interest here is that of a VRML (Virtual Reality Modelling Language) file [36]. VRML97 is an ISO standard specifically designed for distributing 3-D models via the World Wide Web. Many free VRML viewers are available [46]. These viewers, which can function either as stand-alone programmes or as plug-ins for Web browsers, allow users to interact with the 3-D model. Basic interactions include rotating and zooming, which allow the model to be inspected from virtually any viewpoint. More advanced interactions are also possible. The screen shots of VRML models shown here were produced with the Cosmo Player plug-in.

It is also possible to create animations to be viewed by people who do not wish to, or for some reason cannot, install a VRML viewer. One method is to record screen shots of the model as displayed by the VRML viewer and then to use a paint programme to combine the images into an animated GIF file, which should be viewable by any graphical Web browser. Another technique is to use a programme that captures video directly from the screen. The video can then be edited and saved in one of the many available video file formats or, as above, in an animated GIF file.

### 5.9 Quantification of volumes

Once a segmentation has been done, it is relatively easy to extract quantitative information. When a segmentation is done by producing explicit contours, as in the interactive method described above, the enclosed volume can easily be computed when the surface triangulation is performed, in our case by the *Tr3 *programme. The computation is that of the volume of a polyhedron with triangular faces.

When the segmentation is a simple thresholding, the volume calculation is just a matter of counting the selected voxels.

### 5.10 Quantification of intensities

From Fig. 6, for example, it is clear that there is more unoxidized than oxidized lipid. To quantify such an observation, in addition to merely counting pixels with values above some threshold, *Fie *allows the user to compute the *total intensity *in each channel, that is,

Itot=∑i=0255nii
 MathType@MTEF@5@5@+=feaafiart1ev1aaatCvAUfKttLearuWrP9MDH5MBPbIqV92AaeXatLxBI9gBaebbnrfifHhDYfgasaacH8akY=wiFfYdH8Gipec8Eeeu0xXdbba9frFj0=OqFfea0dXdd9vqai=hGuQ8kuc9pgc9s8qqaq=dirpe0xb9q8qiLsFr0=vr0=vr0dc8meaabaqaciaacaGaaeqabaqabeGadaaakeaacqWGjbqsdaWgaaWcbaGaemiDaqNaem4Ba8MaemiDaqhabeaakiabg2da9maaqahabaGaemOBa42aaSbaaSqaaiabdMgaPbqabaGccqWGPbqAaSqaaiabdMgaPjabg2da9iabicdaWaqaaiabikdaYiabiwda1iabiwda1aqdcqGHris5aaaa@4010@

where *i *is the intensity, ranging from 0 to 255, and *n*_*i *_is the number of pixels having intensity *i*. This can be done for one slice or for all slices at once, and for an entire image or for a specified region of interest, such as an individual cell.

To visualize the distribution of relative intensities, one can view the combined red and green channels in the conventional way, by adding reds and greens to make shades of yellow, but this is difficult to quantify. An alternative strategy is to display the difference between the red-channel intensity and the green-channel intensity for each pixel in the image, as in Fig. 8(b). The pixels are shown as grey when the intensity difference is zero, as shades of yellow when the red intensity is greater than the green intensity, and as shades of blue when the opposite is true.

## Competing interests

The authors declare that they have no competing interests.

## Authors' contributions

The 3-D software was developed by WRJF, who also wrote the related text. Biological experiments with live cells and their imaging were done by DM, who provided confocal images for 3-D reconstruction. Overall text on the relevance of 3-D reconstructions for cell biology was written by DM. Both authors take full responsibility for the accuracy of statements and they worked together on finalizing the text.

## Supplementary Material

Additional file 1 3-D model shown in Fig. 2. This file can be viewed with any VRML97 viewer, as discussed in the text. Click here for file

Additional file 2 Animation of 3-D model shown in Fig. 2. This animation was created as a screenshot of the model displayed by the VRML viewer Cosmo Player. The animation should be viewable with any graphical Web browser. Click here for file

Additional file 3 3-D model shown in Fig. 3. Click here for file

Additional file 4 Animation of 3-D model shown in Fig. 3. Click here for file

Additional file 5 3-D model shown in Fig. 4. Click here for file

Additional file 6 3-D model shown in Fig. 5 (top). Click here for file

Additional file 7 3-D model shown in Fig. 5 (bottom). Click here for file

Additional file 8 3-D model shown in Fig. 7. Click here for file
