# Supplementary figures and images for: Three-dimensional reconstruction of cell nuclei, internalized quantum dots and sites of lipid peroxidation
Source: J Nanobiotechnology. 2006 Oct 20;4:10. doi: 10.1186/1477-3155-4-10 (PMC1635722; doi:10.1186/1477-3155-4-10)

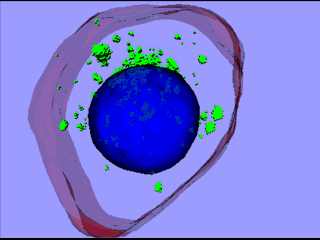

Supplement: Additional file 2 — Animation of 3-D model shown in Fig. 2. This animation was created as a screenshot of the model displayed by the VRML viewer Cosmo Player. The animation should be viewable with any graphical Web browser. [file 1477-3155-4-10-S2.gif]

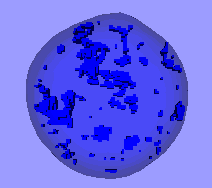

Supplement: Additional file 4 — Animation of 3-D model shown in Fig. 3. [file 1477-3155-4-10-S4.gif]
